# Supplementary material for: Clonal dynamics towards the development of venetoclax resistance in chronic lymphocytic leukemia
Source: Nat Commun. 2018 Feb 20;9:727. doi: 10.1038/s41467-018-03170-7 (PMC5820258; doi:10.1038/s41467-018-03170-7)
Supplement: Supplementary file 1 — Supplementary Information [file 41467_2018_3170_MOESM1_ESM.pdf]

Supplementary Information

	Age (at start of venetoclax)	Number of pretreatments	First diagnosis to start of venetoclax (months)	Months on venetoclax	Maximum dose level reached (mg qod)	Best response	Time to progression (months)	Richter transformation	Time to retreatment (months)	Time to final follow up (months)	Survival status at final follow up
C548	72	2	18.3	5	400	SD	4.5	Yes	5.4	5.6	dead
C577	61	2	104.5	11	400	PR	10.6	No	11.8	33.3	alive
C586	71	8	139.9	14.3	400	SD	14.5	Yes	14.5	18.6	dead
C626	63	1	69.9	17.3	400	PR	16.4	No	17.4	36	alive
C651	61	2	164.1	18.9	400	PR	18.1	No	18.9	35.2	alive
C789	62	2	139.5	3.8	400	PR	3.7	Yes	5.6	6.1	dead
C811	47	2	77.6	22.3	400	PR	21.8	Yes	22.3	34.4	dead
C812	77	3	34.8	22.8	400	PR	21.8	No	23.3	31.4	alive

Supplementary Table 1: Overview of clinical information for all 8 patients treated with venetoclax.

Patient ID	Sample	Purity	Ploidy	Enrichment Kit
C548	T0_PB	0.94	1.97	NimbleGen SeqCap
C548	T1_PB	0.91	3.69	NimbleGen SeqCap
C577	T0_PB	0.97	1.99	NimbleGen SeqCap
C577	T1_LN	0.55	2.00	NimbleGen SeqCap
C577	T2_LN	0.95	1.94	NimbleGen SeqCap
C586	T0_PB	0.94	1.98	NimbleGen SeqCap
C586	T1_PB	0.94	4.01	NimbleGen SeqCap
C586	T2_PB	0.88	4.01	NimbleGen SeqCap
C586	T3_BI	0.66	2.02	NimbleGen SeqCap
C586	T4_BM	0.97	4.05	Agilent Sureselect
C626	T0_PB	0.99	1.98	Agilent Sureselect
C626	T1_PB	0.98	1.97	Agilent Sureselect
C626	T2_LN	0.77	1.96	Agilent Sureselect
C651	T0_PB	0.89	2.00	Agilent Sureselect
C651	T1_LN	0.88	1.94	Agilent Sureselect
C789	T0_PB	0.41	1.99	Agilent Sureselect
C789	T1_LN	0.56	1.98	Agilent Sureselect
C811	T0_PB	0.99	1.99	Agilent Sureselect
C811	T1_LN	0.43	2.39	Agilent Sureselect
C812	T0_PB	0.95	1.94	Agilent Sureselect
C812	T1_PB	0.45	1.94	Agilent Sureselect

Supplementary Table 2: Purity ploidy estimates from sequencing data. The last column indicates which exon capture kit was used for which sample.

C812: before venetoclax therapy

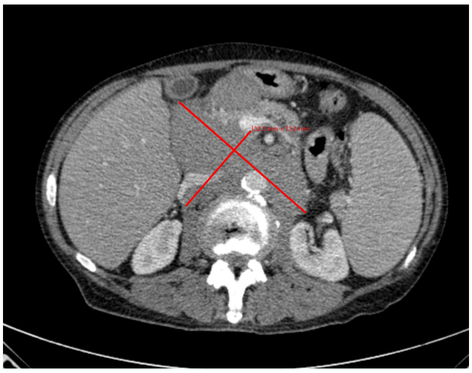

C812: at therapy response

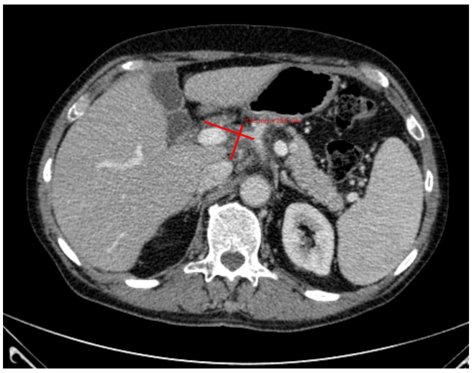

Supplementary Figure 1: Computer tomography (CT) scans of the abdomen from patient C812 at baseline (T0) and month 8 (CT2). Selected CT levels (red lines) demonstrate the maximum diameter of the retroperitoneal tumor bulk at baseline (74 x 132 mm) and of the remaining disease at the time of best response (28 x 44 mm).

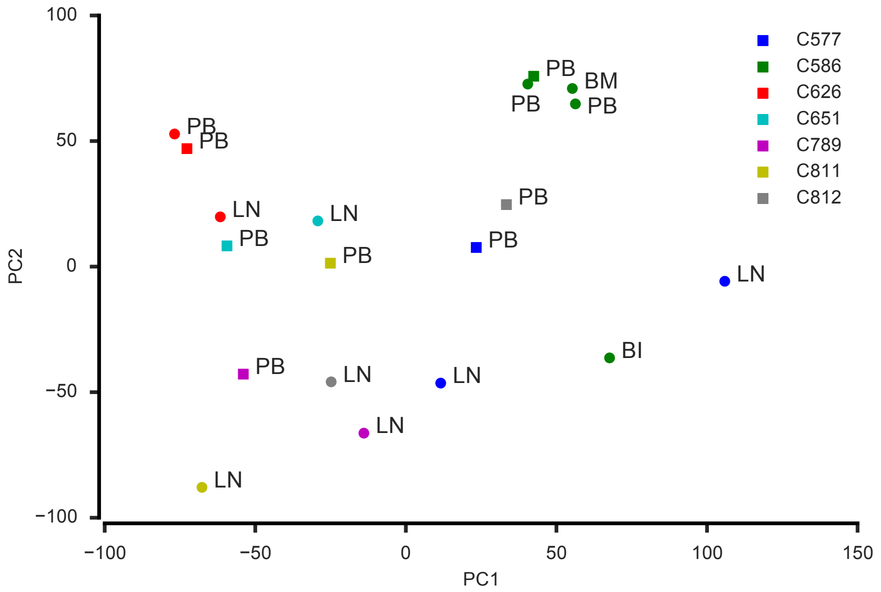

Supplementary Figure 2: Projection of methylation patterns on the first and second principle components, PC1 and PC2 respectively. Methylation patterns of different samples from a patient, including venetoclax-untreated (rectangular symbols) and –treated (circular symbols) samples, are clustered in close proximity. This suggests that the methylation pattern remained stable during venetoclax therapy. Variations are mostly between the different compartments (peripheral blood, lymph nodes, etc.) since contaminations of other cells in the compartment mask the signal of the CLL cancer cells.

a

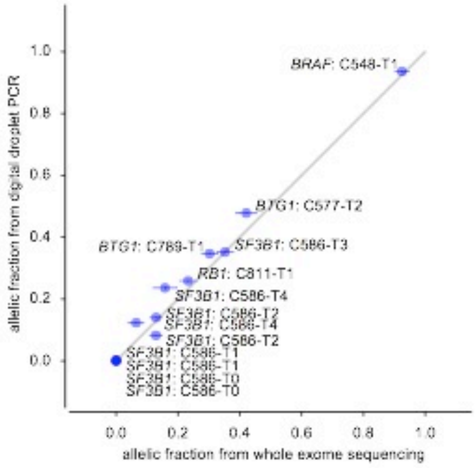

b

***NOTCH1*: c.7541_7542delCT; p.P2514fs**

Sample	ddPCR mutant DNA count	ddPCR wild type DNA count	Allelic fraction	Comment
C548-T0-PB	N/A	N/A	N/A	no material for ddPCR
C548-T1-PB	4960	120	0.976	
C577-T0-PB	2480	2500	0.498	
C577-T1-LN	1728	3880	0.308	
C577-T2-LN	10680	10520	0.504	
C626-T0-PB	2600	1230	0.679	
C626-T1-PB	2540	2100	0.547	
C626-T2-LN	1792	1732	0.509	
C811-T0-PB	15	78520	0.00019	
C811-T1-LN	774	4260	0.154	

c

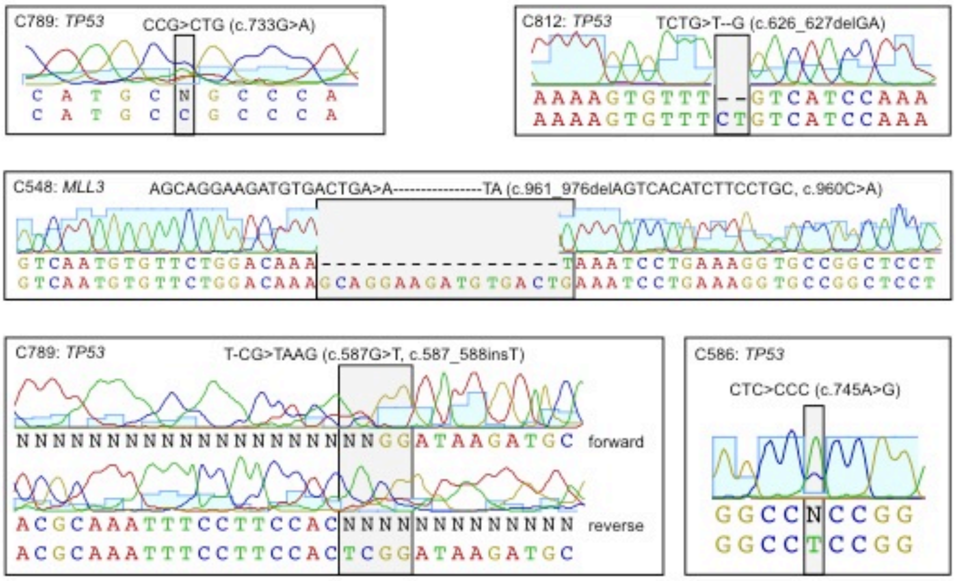

Supplementary Figure 3: Validation of key point mutations highlighted in **Fig. 1b**. (**a**) A comparison of allelic fractions of a set of single nucleotide mutations from digital droplet PCR with whole exome sequencing. Error bars show the standard deviation and were determined from whole exome sequencing. (**b**) Validation of *NOTCH1* frame shift deletions using digital droplet PCR. (**c**) Validation of the remaining mutations with conventional dideoxy sequencing that were not detected previously as part of a study inclusion criterion[16]. Note that only the *BIRC3* mutation in sample C548 could not be validated due to a lack of material.

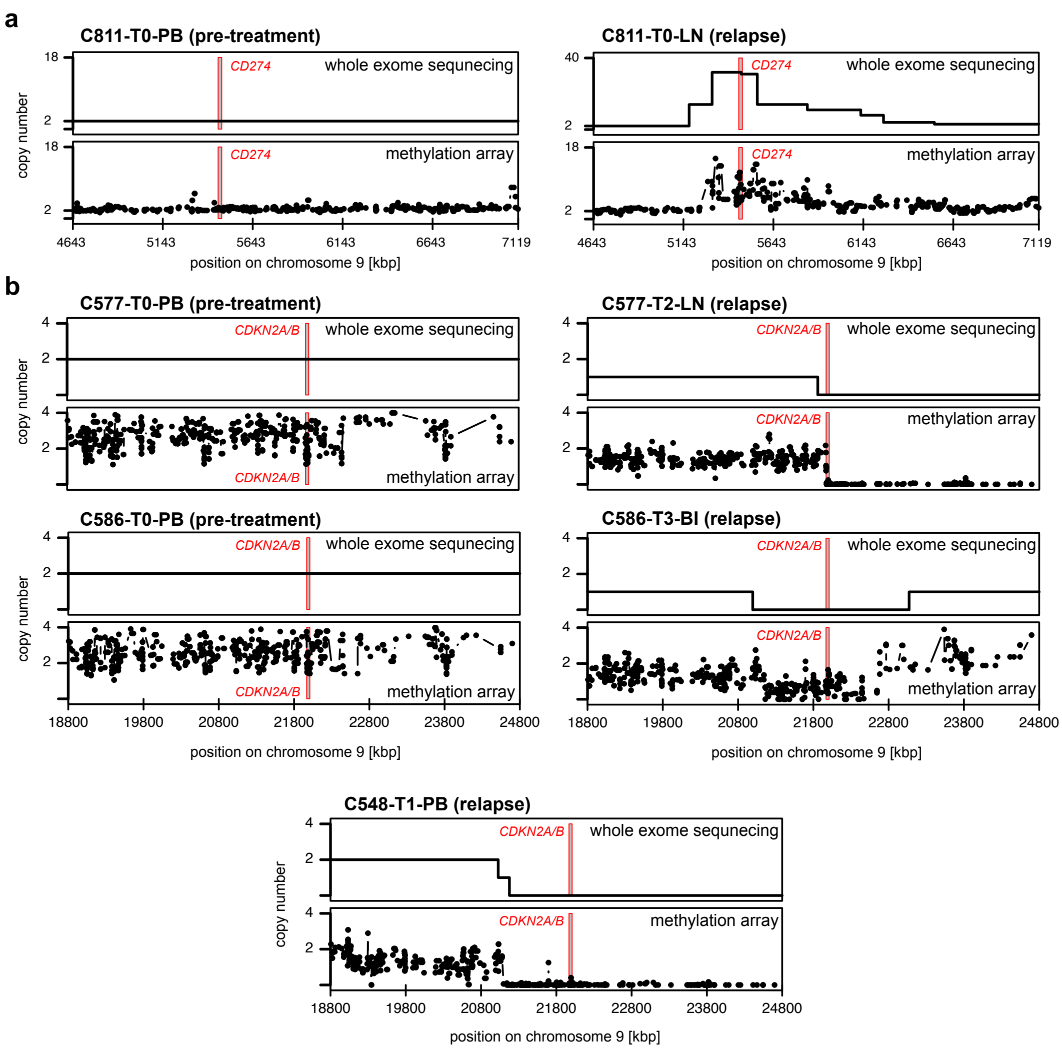

Supplementary Figure 4: Validation of whole exome sequencing-derived copy number alterations in: (**a**) *CD274* (PD-L1) and (**b**) *CDKN2A/B* using methylation arrays. The pre-treatment case of patient C548 was not available due to a lack of sufficient material.

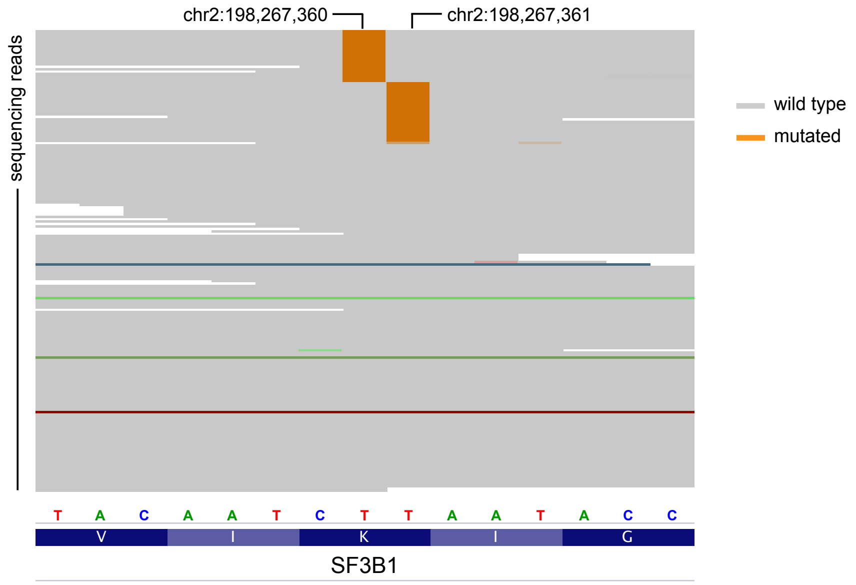

Supplementary Figure 5: Sequencing reads harboring two *SF3B1* mutations (c.1996A>C; p.K666Q and c.1997A>C; p.K666T) in C586. The mutations are affecting the same codon but evolved in two independent clones during the treatment, as they appear on independent sequencing reads.

a

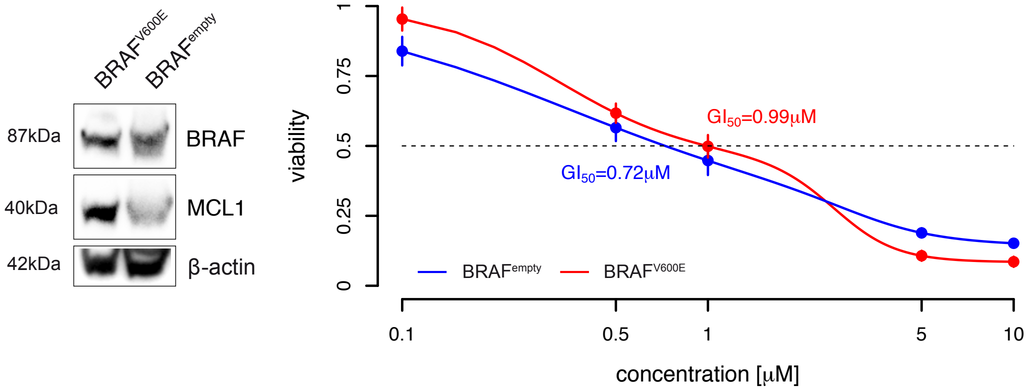

b

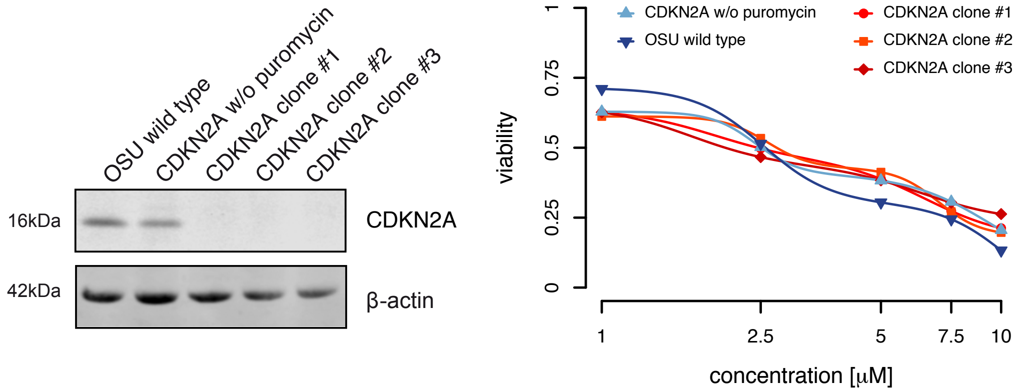

Supplementary Figure 6: Overexpression of oncogenic *BRAF* and knockdown of *CDKN2A*. (**a**) Western blot analysis of BRAF and MCL1 in the *BRAF*^{V600E} overexpressing U-2932 cell line versus its empty vector control. Both, the *BRAF*^{V600E}-transduced and the empty vector control U-2932 cell lines were screened against increasing concentrations of venetoclax. (**b**) To validate a successful *CDKN2A* knockout via CRISPR/Cas9 western blotting was performed. For this purpose wildtype OSU cells, lentivirally transduced but not puromycin-selected OSU cells and potential knockout clones after selection were lysed and an immunoblot against CDKN2A and ß-actin was performed after 60 µg of the lysate were loaded per lane. These same clones were employed to investigate, whether the *CDKN2A* knockout impacts on sensitivity towards venetoclax. Therefore these clones were incubated with increasing concentrations of venetoclax. The amount of apoptotic cells was determined by flow cytometry (Annexin V/7AAD negative fraction). This experiment has been performed two times with similar results. One representative result is shown here.

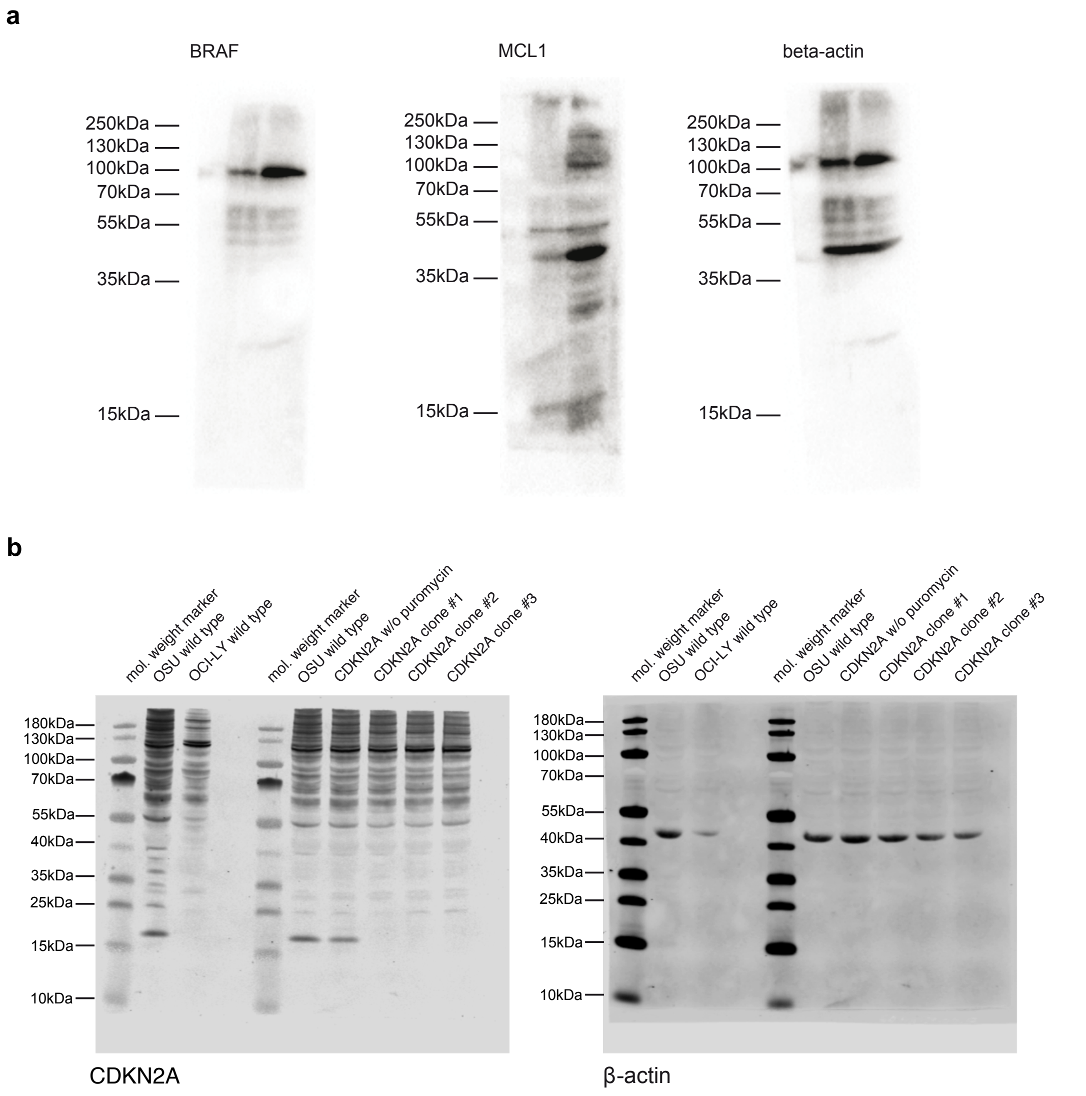

Supplementary Figure 7: Full-length images of most important western blots. (**a**) western blots shown in **Fig. 3**. (**b**) Full western blots of **Supplementary Fig. 6b**. Please note, that the repetition of OSU and OCI-LY wild type (lane 2 and 3) are not shown in **Supplementary Fig. 6b** because they are not relevant in this context.
