# Supplementary material for: Clonal dynamics towards the development of venetoclax resistance in chronic lymphocytic leukemia
Source: Nat Commun. 2018 Feb 20;9:727. doi: 10.1038/s41467-018-03170-7 (PMC5820258; doi:10.1038/s41467-018-03170-7)
Supplement: Supplementary file 3 — Description of Additional Supplementary Files [file 41467_2018_3170_MOESM3_ESM.docx]

**Description of Additional Supplementary Files**

File Name: Supplementary Data 1

Description: Assignment of somatic mutations to their corresponding clonal populations shown in Fig. 2, all detected somatic mutations, copy number states, and subclonal copy numbers.
